# High-Performance Prediction of Human Estrogen Receptor Agonists Based on Chemical Structures

**DOI:** 10.3390/molecules22040675

**Published:** 2017-04-23

**Authors:** Yuki Asako, Yoshihiro Uesawa

**Affiliations:** Department of Clinical Pharmaceutics Meiji Pharmaceutical University, 2-522-1 Noshio, Kiyose, Tokyo 204-8588, Japan; y121007@std.my-pharm.ac.jp

**Keywords:** machine learning, random forest, estrogen receptor, Tox21 data challenge 2014, QSAR prediction model

## Abstract

Many agonists for the estrogen receptor are known to disrupt endocrine functioning. We have developed a computational model that predicts agonists for the estrogen receptor ligand-binding domain in an assay system. Our model was entered into the Tox21 Data Challenge 2014, a computational toxicology competition organized by the National Center for Advancing Translational Sciences. This competition aims to find high-performance predictive models for various adverse-outcome pathways, including the estrogen receptor. Our predictive model, which is based on the random forest method, delivered the best performance in its competition category. In the current study, the predictive performance of the random forest models was improved by strictly adjusting the hyperparameters to avoid overfitting. The random forest models were optimized from 4000 descriptors simultaneously applied to 10,000 activity assay results for the estrogen receptor ligand-binding domain, which have been measured and compiled by Tox21. Owing to the correlation between our model’s and the challenge’s results, we consider that our model currently possesses the highest predictive power on agonist activity of the estrogen receptor ligand-binding domain. Furthermore, analysis of the optimized model revealed some important features of the agonists, such as the number of hydroxyl groups in the molecules.

## 1. Introduction

Estrogen receptors (ER) belong to the steroid receptor superfamily of ligand-dependent transcription factors [1]. Compounds related to ER activation, such as isoflavones and polycyclic aromatic hydrocarbons, disrupt the endocrine processes in humans and other species, severely affecting reproduction and growth [2,3]. Therefore, screening for ER agonists can counteract environmental contaminants and improve public health. Although ultra-high-throughput screening systems have been developed for several adverse-outcome pathways [4], experimental in vitro detection is limited by the vast number of screening targets. Consequently, a comprehensive assay is precluded by both economics and time. In contrast, predictive methods based on chemical structures can greatly accelerate the estimation and are expected to replace wet experimental systems. 

The National Institute of Health (NIH), Environmental Protection Agency, and Food and Drug Administration have collaborated to launch the Tox21 challenge, a large project targeting a variety of toxicity problems, including the environmental effects of ER agonists [5,6]. The Tox21 project has assayed the activities of the compounds in the Tox21 10 K library [4], which contains 10,000 chemicals for toxicity estimation. The adverse-outcome pathways selected for toxicity evaluations are the androgen receptor (AR), aryl hydrocarbon receptor (AhR), estrogen receptor (ER), aromatase and peroxisome proliferator-activated receptor (PPAR), nuclear factor (erythroid-derived-2)-like 2/antioxidant responsive element (ARE), ATP-ase family AAA domain containing 5 (ATAD5), heat shock factor response element (HSE), mitochondrial membrane potential (MMP), and p53 [7].

One aim of the Tox21 project is to construct a predictive system based on computational toxicology. The Tox21 Data Challenge 2014 was organized by NIH’s National Center for Advancing Translational Sciences as a “cloud-sourcing” search for high-performance predictive models of adverse-outcome pathways. Using computational toxicology technologies, participants competed in accuracy of predictive models with the biological toxic responses of compounds in the Tox21 10 K compound library, whose activities were known, applying the abovementioned adverse-outcome pathways as the training set [8]. Dr. Yoshihiro Uesawa, a winner of the 2014 challenge and a coauthor of the present study, constructed a predictive model for the ER-ligand binding domain (ER-LBD), which showed the best performance among the models submitted by the registered teams [9]. On the contrary, there is a large-scale modeling project, Collaborative Estrogen Receptor Activity Prediction Project (CERAPP), in which many groups built and evaluated ER QSAR models [10]. Multiple models were developed through collaboration between 17 international research groups with a common training set of 1677 chemicals, resulting in 40 prediction models for binding, agonist, and antagonist ER activity. External validation was performed with 7522 chemicals from the literature. This project demonstrated that using a consensus of different models with large-scale data set allows for the improvement of prediction abilities. In particular, the consensus model reached a balanced accuracy of >0.9 (using high-quality data).

According to the comprehensive review of Chou’s five-step rule [11], which has been implemented in various publications [12,13,14,15,16], the following rules are useful for developing a statistical predictor for a biological system: (i) construct/select a valid dataset for training and testing the predictor; (ii) translate biological sequences into numerical descriptors that truly reflect the effectiveness of target classes; (iii) select/develop an intelligent operational algorithm; (iv) correctly perform cross-validation tests that objectively evaluate the expected outcomes of the predictor; and (v) construct a web predictor for the model that is accessible to the public.

Uesawa’s ER model was based on a machine learning method called random forest (RF). RF is an ensemble learning method that decides the best predictive result by majority rule of the various predictive results gained from many decision trees [17,18,19]. Each tree is constructed from bootstrapped data of the training set. This method achieves high predictive performance at low computational cost, even when the dataset is large or prejudiced. It also assesses the importance of the variables used in the construction. In the previous study, we confirmed that rigorous selection from many kinds of RF models significantly improved the performance of RF. However, we also observed an overfitting tendency [9]. In this study, we attempt to improve the predictive performance of the previous RF construction by a novel RF optimization technique.

## 2. Methods

### 2.1. Conformations and Descriptors

Various descriptors were calculated by optimizing the three-dimensional structures of their chemical constituents, as implemented in the Tox21 Data Challenge 2014 [9]. Briefly, the SD file of each descriptor, which contains the chemical structure and ER-LBD assay results (active or inactive) of the compound, was downloaded from a homepage dedicated to the competition [8]. The three-dimensional conformations of the chemical structures in two configurations (a charged form at neutral pH and an uncharged form) were calculated in the Molecular Operating Environment (MOE) 2013.08 (Chemical Computing Group Inc., Montréal, QC, Canada) [20]. Finally, 4071 different molecular descriptors were generated by MOE, MarvinView 6.0.0 (ChemAxon Kft., Budapest, Hungary) [21], and Dragon 6 (Talete srl., Milano, Italy).

### 2.2. Construction of Predictive Models

**Figure 1:**
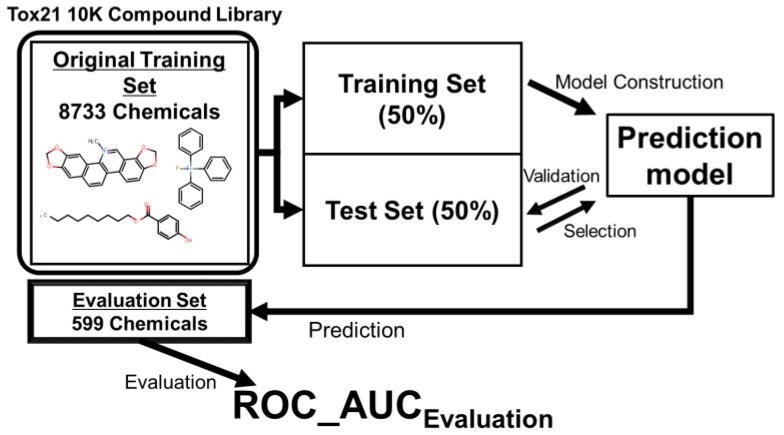
Scheme of the model construction.

In the previous study [9], the original training set of 8733 chemicals and final evaluation set of 599 chemicals were downloaded from the homepage of the Tox21 Data Challenge website [8]. The same data were used in the present study. The original training set was randomly divided into a training set (50%) for constructing the predictive model and a test set (50%) for model validation. The selection processes in the predictive models were constructed from the training set (50%). In our basic method, we applied the bootstrap-forest function in JMP Pro 12.0.1 (SAS Institute Inc., Cary, NC, USA) as the RF algorithm [22]. Finally, the compounds in the final evaluation set were predicted by the model. The evaluation indices were the predictive ability of each model in the evaluation step, and the area under the receiver operating characteristic curve (ROC_AUC Evaluation) (see Figure 1).

Different methods in statistical prediction, such as the n-fold cross-validation test, sub-sampling test, independent dataset test, and jackknife cross-validation test, have been adopted for evaluating the performance of a prediction model [23,24,25,26]. As the jackknife test can lead to unique results [27,28], it has been widely used in bioinformatics [25,29,30,31,32,33,34]. In this study, for saving computational time, the independent data test was used to investigate the performance of the prediction model.

### 2.3. Effects of Descriptors

**Figure 2:**
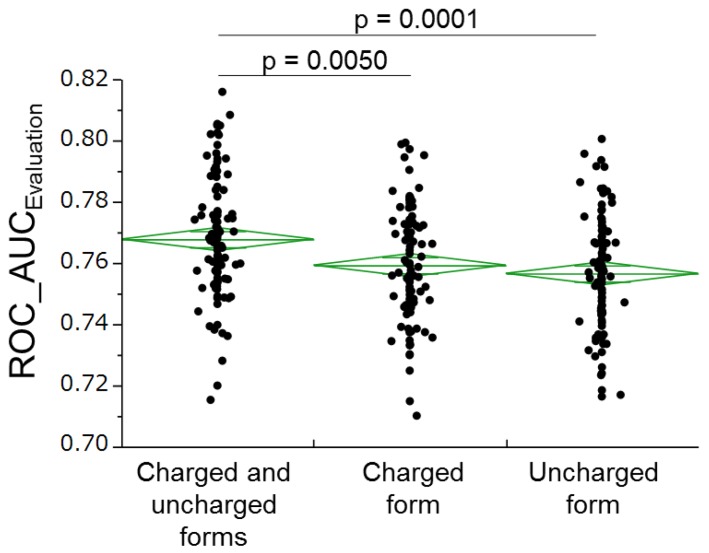
Charged and uncharged forms 100 random forest (RF) models were constructed for the charged, uncharged, and both forms of each descriptor. All models were involved in predicting the activities of the estrogen receptor ligand-binding domain for the compounds in the final evaluation set. 100 ROC_AUC values were plotted for each group. Green lines denote the averages and their 95% confidence intervals.

MOE computed 369 charged and 369 uncharged forms of each molecular descriptor (738 descriptors in total). For each descriptor group (charged, uncharged, and total), we constructed 100 RF models and compared their average ROC-AUC evaluation values. From the results, we estimated the contributions of the charged and uncharged forms in the predictive performance (Figure 2).

#### Number of Descriptors

**Figure 3:**
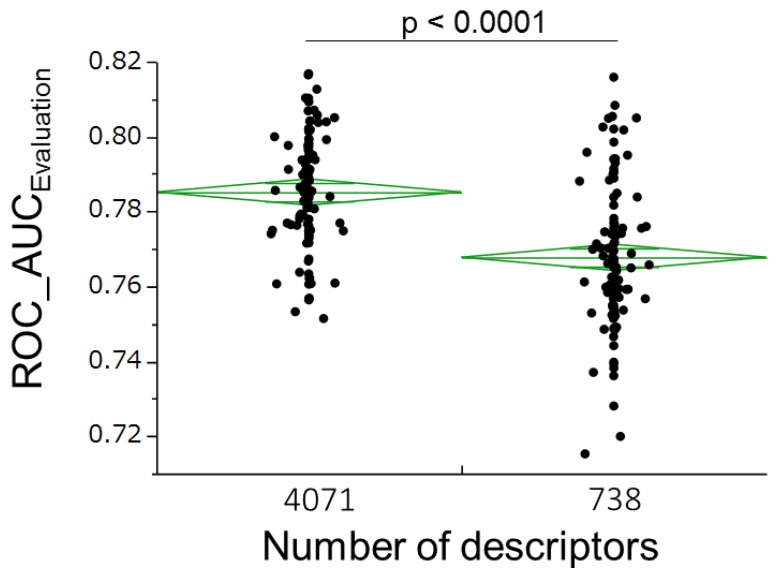
Number of descriptors 100 RF models were constructed for both numbers of descriptors. All models were involved in predicting the activities of estrogen receptor ligand-binding domain for compounds in the final evaluation set. 100 ROC_AUC values were plotted for each group. Green lines denote the averages and their 95% confidence intervals.

To ascertain whether the performance of our model would be improved by adding more descriptors, we constructed 100 RF models of the 4071 descriptors calculated by MOE, MarvinView, and Dragon, and compared their predictive abilities with those of RF models constructed from MOE descriptors (738 kinds) alone (Figure 3).

### 2.4. Effects of Hyperparameters

**Figure 4:**
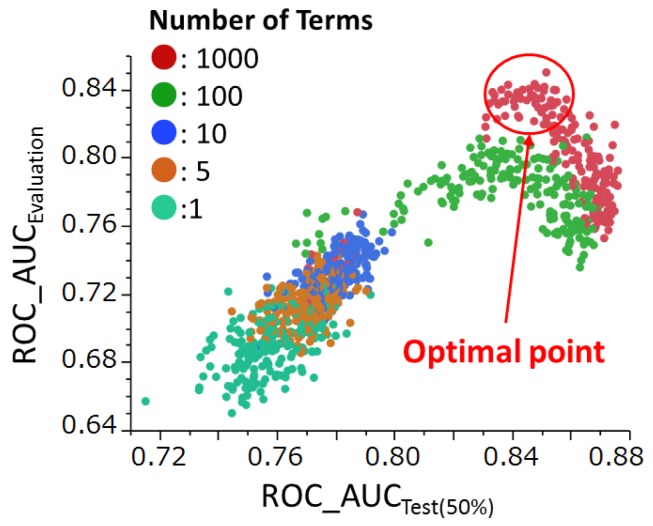
Relationship between ROC_AUC values in models constructed from the test set (50%) and the final evaluation set. Each point denotes the performance of the model. This figure is referred from [9].

To find the best combination of hyperparameters in the RF construction, we scanned two hyperparameters under the following conditions (10 iterations per condition): Number of Terms (the number of columns considered as splitting candidates at each split (range 1–1000)) and Maximum Splits per Tree (the maximum number of splits for each tree (range 2–400)). For each Number of Terms we constructed 190 models. The ROC_AUC values of all constructed models were then estimated on the compounds in the test set (50%) and evaluation set (Figure 4). 

In Figure 4, a total of 950 RF models are shown. These models are part of the modeling results. We generated 100 models more with a better combination of the hyperparameters, and a final model was selected from all the models based on the ROC_AUC values. This strategy for RF model construction was reported as “Rigorous Selection,” which indicated an excellent performance in the Tox21 data challenge 2014 [9]. 

**Figure 5:**
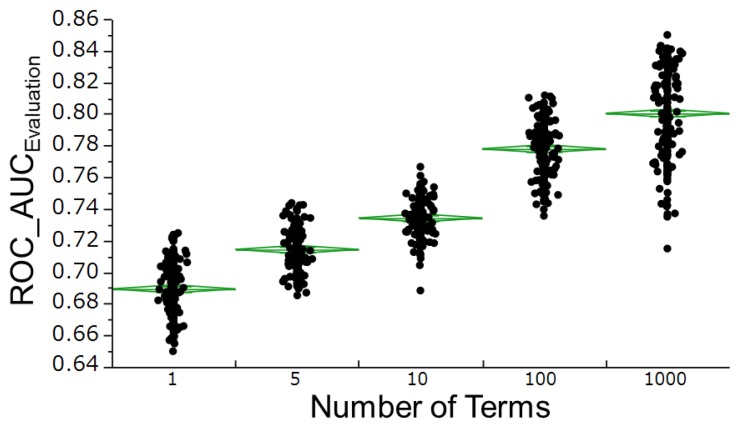
Effects of the hyperparameter Number of Terms on the RF modeling 190 RF models were constructed in each group, and all models were then involved in predicting the activities of the estrogen receptor ligand-binding domain for compounds in the final evaluation set. Plotted are the ROC_AUC values for the final evaluation set in each group. Green lines denote the averages and their 95% confidence intervals.

**Figure 6:**
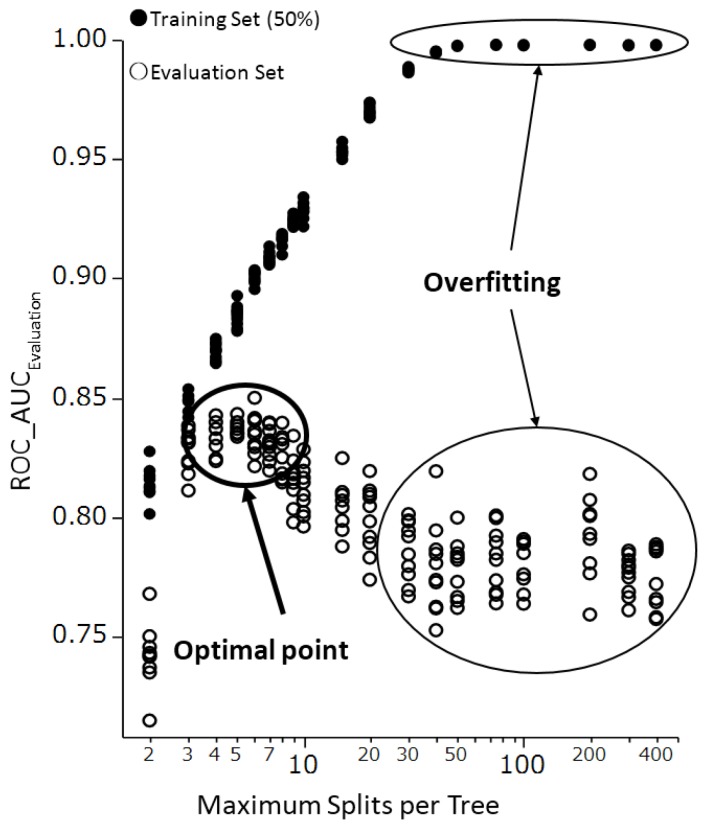
Effects of the hyperparameter Maximum Splits per Tree on the RF modeling ROC_AUC values of the training set (50%) and final evaluation set are plotted in closed and open circles, respectively. Large Maximum Splits per Tree introduced model overfitting. The predictive ability was optimized for Maximum Splits per Tree = 6.

Furthermore, the 190 ROC_AUC Evaluation values obtained for each Number of Terms were compared (Figure 5). Finally, for the models with Number of Terms = 1000, we generated scatter plots between Maximum Splits per Tree and the ROC_AUCs in the training set (50%) and evaluation set (Figure 6).

### 2.5. Statistical Treatment

Significant differences in the means were tested by an unpaired Student’s t-test and one-way ANOVA, followed by least-significant difference analysis. All analyses, including the ROC_AUC calculations, were performed in JMP-Pro. The significance level was set to *p* < 0.05.

## 3. Results and Discussion

### 3.1. Effects of Descriptors

Integrated descriptors from both charged and uncharged forms improved the predictive ability of the RF models, relative to descriptors from unilateral forms (Figure 2). Varying the charge conditions increased the diversity of the information in the RF models. Increasing the number of descriptors from 738 (calculated by MOE alone) to 4071 (calculated by three software programs, MOE, Dragon and MarvinView) also improved the predictive ability of the RF models (Figure 3). On the other hand, feature selections based on the known importance of each descriptor during the RF-modeling failed to improve the model performance (data not shown). These observations suggest that a large number of descriptors are advantageous for our models’ performance.

### 3.2. Effects of Hyperparameters

The AUC values in the test set (50%) and the final evaluation set were not simply correlated; rather, there was an optimal point at which the AUC of the test set (50%) corresponded to the highest AUC of the evaluation model (Figure 4). This point was emphasized in our previous paper [9]. In the competition, model selection was optimized using the AUC values in the test set (50%) because the AUCs between the test set (50%) and the training set (50%) showed good linear correlation [9]. Next, we analyzed the true performance of our models on the final evaluation set [9] (see Figure 4).

All further investigations in the current study were performed on the final evaluation set. In a scanning search for the Number of Terms hyperparameter, the best predictive model was obtained at Number of Terms = 1000 (Figure 5). A scanning search for the predictive capabilities of Maximum Splits per Tree under restricted conditions, with Number of Terms = 1000, was also performed (Figure 6). Figure 4 shows the scatter plot of ROC_AUC values in models with different combinations of hyperparameters such as Number of Terms and Maximum Splits per Tree. The best performance was among models with Number of Term = 1000 (red points). However, the red points include different values of Maximum Splits per Tree between 2 and 400. Therefore, the data of the red points were selected and re-plotted in Figure 6 with Maximum Splits per Tree in horizontal axis to display the relation between the prediction performance and the hyperparameter.

RF models with high Maximum Splits per Tree were found to overfit the training set. The optimal predictive ability of the models was obtained for Maximum Splits per Tree = 6 (Figure 6). We inferred that we could regulate the overfitting by lowering the optimal point of Maximum Splits per Tree; that is, by restricting the tree growth.

### 3.3. Discrimination Potential of Improved Models

**Figure 7:**
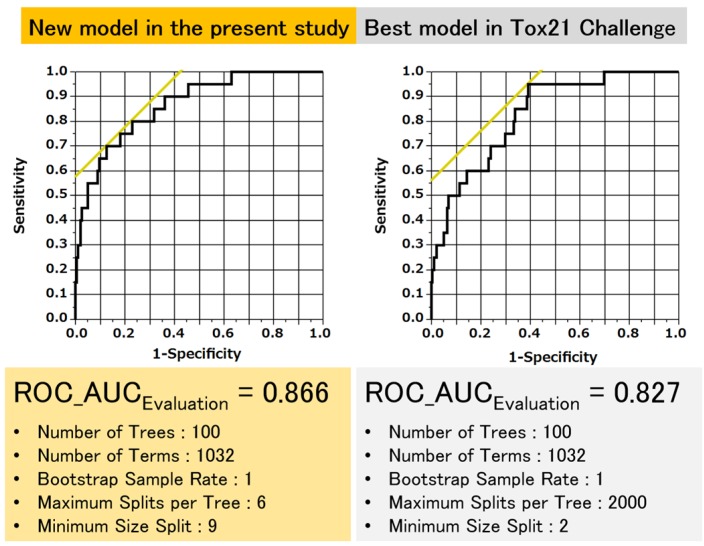
ROC curves for predicting ER-LBD-activating compounds with the newly proposed model (left) and the best model of the Tox21 Data Challenge 2014 ROC-AUCs and hyperparameter values in the models are also described.

Figure 7 shows the ROC curves that evaluate the discrimination capacities of the new predictive model and the best model in the Tox21 Data Challenge 2014. The ROC-AUC values for the compounds with ER-LBD activities in the final evaluation test set were 86.6% and 82.7% in the present and previous models, respectively. In addition, the ROC between sensitivity and 1-specificity was better balanced in the present model than in the previous model. Overall, the predictive performance of the present model surpasses that of the previous model. 

Figure 7 ROC curves for predicting ER-LBD-activating compounds in the newly proposed model (left) and the previous best model in the Tox21 Data Challenge 2014 ROC-AUCs and hyperparameter values in the models are also described.

### 3.4. Most Important Descriptors

The importance of the descriptors used in constructing the present model was calculated from their split rankings (count of each descriptor usage when partitioning the decision trees in the RF modeling) and G2 values (chi-squared values of the likelihood-ratios) [35]. The descriptor rankings in 100 RF models under the optimized hyperparameter conditions are listed in Table 1. The top-ranking ER-LBD activator was SpMin 1-Bh(m). The topological shape, nArOH and number of OH groups bound to the aromatic rings might contribute to the activity of this compound in the ER-LBD assay [36].

## 4. Conclusions

We constructed a new predictive model with higher discrimination ability for estrogenic compounds than our previous best model, which was submitted to the Tox21 Data Challenge 2014. It means that we had to succeed to deliver the excellent predictive power on estrogenic compounds. Correlating the results of the ER-LBD sub-challenge in Tox21 data challenge 2014 with our results, we believe that the current model, which uses a simple model based on ROC_AUC as an evaluation criterion, possesses the best prediction ability for ER-LBD agonist activity. The best conditions for the RF modeling regulated the overfitting of the test set (50%). This regulation was found to be important in the RF model constructions. Furthermore, the model analyses revealed that in compounds such as SpMin 1-Bh(m), interactions with ER were facilitated by the structural and physicochemical properties of the compounds and the number of phenolic OH groups. This modeling approach will be useful for predicting the toxicity of compounds and producing new drugs and chemicals. As user-friendly and publicly accessible web servers represent the future on developing practical and more useful models, we shall work in the near future on providing a web server for the method presented in this paper.

## Figures and Tables

**Table 1:** Most important descriptors Listed are the top 10 ranked descriptors in the RF modeling, determined from the split and G2 rankings.

Descriptor	Meaning	Software	State	Sprit	G2	Sprit Ranking	G2 Ranking
SpMin1_Bh(m)	smallest eigenvalue n. 1 of Burden matrix weighted by mass	Dragon	Uncharged	28.1	38.5	1	1
SpMin1_Bh(m)	smallest eigenvalue n. 1 of Burden matrix weighted by mass	Dragon	Charged	17.2	21.1	2	2
SpMin1_Bh(s)	smallest eigenvalue n. 1 of Burden matrix weighted by I-state	Dragon	Uncharged	9.3	11.4	6	3
SpMin1_Bh(i)	smallest eigenvalue n. 1 of Burden matrix weighted by ionization potential	Dragon	Uncharged	5.0	5.7	13	9
nArOH	number of aromatic hydroxyls	Dragon	Charged	17.0	10.5	3	4
nArOH	number of aromatic hydroxyls	Dragon	Uncharged	13.3	7.7	4	6
O-057	phenol / enol / carboxyl OH	Dragon	Charged	13.1	7.8	5	5
Chi_Dt	Randic-like index from detour matrix	Dragon	Charged	5.8	6.1	8	7
CATS2D_03_LL	CATS2D Lipophilic-Lipophilic at lag 03	Dragon	Charged	4.8	6.0	14	8
CATS2D_05_LL	CATS2D Lipophilic-Lipophilic at lag 05	Dragon	Charged	5.6	2.7	9	19
logd(pH = 5.5)	Lipophilicity under pH = 5.5 condition	Marvin	-	5.5	5.7	10	10
vsurf_HB7	H-bond donor capacity 7	MOE	Charged	6.5	3.2	7	17
